# *Streptomyces tamarix* sp. nov.: antagonism against *Alternaria gaisen* producing streptochlorin, isolated from *Tamarix* root soil

**DOI:** 10.3389/fmicb.2023.1273842

**Published:** 2023-11-21

**Authors:** Yi-huang Chen, Yi Zheng Wu, Qin Liu, Zhanfeng Xia, JianMing Wang, Xiao-Xia Luo

**Affiliations:** ^1^Key Laboratory of Protection and Utilization of Biological Resources in Tarim Basin of Xinjiang, Production & Construction Corps, Alar City, China; ^2^School of Life Science and Technology, Tarim University, Alar, China

**Keywords:** *Alternaria gaisen*, streptochlorin, antibacterial mechanism, antagonistic, *Streptomyces*

## Abstract

By the end of 2021, the pear yield in Xinjiang reached 1,795,900 tons, accounting for 1/9 of the country. Pear black spot, caused by *Alternaria gaisen* disease, has had a significant impact on the pear industry. *A. gaisen* can infect nearly all pear plants, resulting in black spots on the fruit that negatively affect both yield and quality. This study focused on the TRM76323 strain of Streptomyces, which was isolated from the soil of *Tamarix chinensis* in Xinjiang Province. Through a multiphase classification and identification method, the genetic classification status of the antagonistic strains was determined. The study also identified the antibacterial active components of streptochlorin using modern isolation and purification techniques. The antagonistic activity of Streptomyces against *Alternaria* was analyzed through *in vitro* and *in vivo* experiments. This research not only expanded the resource bank of antagonistic microorganisms in extreme environments in Xinjiang, but also identified active components that could contribute to the development of new drug lead compounds. Additionally, this study presents a novel approach for the prevention and control of pear black spot disease.

## Introduction

Pear is an important fruit tree across the globe. However, *Alternaria gaisen* disease, known as pear disease, seriously causes a substantial loss in the pear industry. *Alternaria* species are a group of fungi that can cause plant diseases. These fungi, which usually exist in plant residues and living plants, are very harmful to crops. Specifically, they can cause pre- and post-harvest diseases in wheat, corn, celery, pepper, and other vegetables and fruits, including Aksu apples, red dates, and Korla fragrant pears, resulting in considerable crop losses (Chauhan et al., 2009). Currently, the main method for controlling *A. gaisen* involves the application of chemical agents; physical and chemical methods are not effective in controlling the spread of *A. gaisen* disease. Therefore, the prevention and control of microbial-derived biological agents is receiving increasing attention (Dale et al., 2017).

Actinobacteria produce large amounts of bioactive secondary metabolites (Jose et al., 2021). *Streptomyces* was first proposed in the early 1940s (Waksman and Henrici, 1943). So far, the genus *Streptomyces* includes a large number of well-described new species and has the ability to produce strong antibacterial properties (Brown et al., 1976; Ser et al., 2016). They have a wide variety of activities and are commonly used. In addition to their bactericidal, bacteriostatic, anti-infective, and other effects, their properties also enable them to be used as insecticides, anti-tumor agents, herbicides, and immunosuppressants. They are extremely important for various industries, including medicine, health and agriculture while also being applicable for environmental protection (Waksman et al., 1944; Ser et al., 2016). Phuakjaiphaeo et al. (2016) identified antifungal compounds from endophytic *Streptomyces* CEN26 against *Alternaria*. Sharma and Manhas (2022) validated the biological control potential of *Streptomyces* M4 against *Alternaria* leaf spot disease. Separated *Streptomyces lydicus* M01 regulates soil microbial community and alleviates foliar disease caused by *Alternaria* on cucumbers (Wang et al., 2020). Xinjiang, China, contains diverse landforms, such as deserts (e.g., Gobi), lakes, basins, grasslands, mountains, rivers, and swamps. Because of its special geographic location, Xinjiang is exposed to various environmental stresses, including drought, high temperatures, and salinity, which may induce the production of specific antibiotics by microbes.

In this study, a novel *Streptomyces* strain (TRM 76323^T^) was isolated from the soil, after which its broad-spectrum antifungal activities were revealed. The morphological, physiological, and biochemical characteristics of TRM 76323^T^ were analyzed. Moreover, streptochlorin was isolated and purified from the fermentation broth of this strain. The effects of a crude extract on *A. gaisen* mycelial growth, morphology, and cell ultrastructure were examined. In addition, the utility of TRM 76323^T^ as a biocontrol agent was investigated in an *in vitro* experiment. The objective of this study was to identify and charammcterize important actinobacterial resources for controlling fungal phytopathogens. The findings of this study may be useful for protecting agriculturally important plants from diseases.

## Materials and methods

### Isolation of strain TRM 76323^T^

Strain TRM 76323^T^ was isolated from a *Salix rhizosphere* soil sample collected along the Ahe Highway in Xinjiang (36.888 E, 82.553 N; at an altitude of 1,664.8 m). The strain was isolated on glycerol arginine medium (2.00 g arginine, 12.00 g glycerol, 0.50 g MgSO_4_, 1.00 g K_2_HPO_4_, and 17.00 g agar), as previously described (Sun et al., 2020). The pH of the medium was adjusted to 7.5 using Gause's medium. Strain TRM 76323^T^ was grown for 1 week at 30°C.

### Analysis of morphological, cultural, physiological, and biochemical characteristics

To determine the best medium, strain TRM 76323^T^ was cultured on various International Streptomyces Project (ISP) media, including ISP1, ISP2, ISP3, ISP4, ISP5, ISP6, and ISP7 (Xing et al., 2022). Additionally, it was cultured on Gause's medium, potato dextrose agar medium, and nutrient agar medium. The pH of all media was adjusted to 7.0. The strain was grown and maintained on an ISP4 synthetic medium. The JSM-6360 scanning electron microscope (JEOL, Ltd., Tokyo, Japan) was used to examine the cell morphological characteristics of the spores and mycelia on ISP4 medium in plates that were incubated at 28°C for 1 week. Carbon-source utilization tests were conducted following the method described by Law et al. (2017), using the basal medium recommended by Pridham (Gottlieb, 1948). The growth ability of the strain TRM 76323^T^ was assessed at different temperatures ranging from 4 to 50°C (4, 12, 15, 20, 25, 28, 30, 37, 40, 45, and 50°C) and pH levels ranging from 4 to 11 (pH 4, 5, 6, 7, 8, 9, 10, and 11). The study aimed to determine the tolerance of the system to different concentrations of NaCl (0, 5, 10, 15, 20, and 25%; w/v). Additionally, the production of peroxidase, urease, esterase, and catalase was analyzed following the methods described by Gerhardt et al. (1994). The study also investigated the use of a sole carbon source (0.5%; w/v), cellulose decomposition, starch hydrolysis, liquefaction of gelatin, milk peptization and solidification, nitrate reduction, and H_2_S production.

### Chemotaxonomic features

The types of amino acids in cell wall hydrolysates and the whole-cell sugar contents were determined (Tindall et al., 2007). Polar lipids were identified *via* two-dimensional thin-layer chromatography involving 10% ethanolic molybdophosphoric acid, as described by Minnikin et al. (1984).

### 16*S rRNA* gene and multi-locus sequence analyses

Strain TRM 76323^T^ was identified through the analysis of its morphological, physiological, and biochemical characteristics. The molecular identification of the strain was performed by amplifying the 16*S rRNA* gene sequence using the prokaryotic universal primers 27F (5′-AGTTTGATCMTGGCTCAG-3′) and 1492R (5′-GGTTACCTTGTTACGACTT-3′; Qi et al., 2019). The following housekeeping genes used in a previous *Streptomyces* multi-locus sequence analysis were selected: *atpD* (ATP synthase, beta subunit), *gyrB* (DNA gyrase B subunit), *recA* (recombinase A), *rpoB* (RNA polymerase, beta subunit), and *trpB* (tryptophan synthase, beta subunit). The average nucleotide identity (ANI) value was calculated as described by Lee et al. (2016). The digital DNA–DNA hybridization value was calculated according to formula 1 described by Meier-Kolthoff et al. (2013), which is available on the Genome-to-Genome Distance Calculator website. The strain TRM76323^T^ draft genome sequence was analyzed using the Illumina platform. ABYSS software was used to assemble the sequencing data (Alanjary et al., 2019). The antiSMASH program (version 6.0) was used to detect biosynthetic gene clusters predicted to produce secondary metabolites (Blin et al., 2021). The Comprehensive Antimicrobial Research Database (CARD) was used to predict resistance genes (https://card.mcmaster.ca/analyze/rgi).

### Fermentation and purification of TRM 76323^T^

Strain TRM 76323^T^ from the ISP4 culture medium was inoculated into 1 L shake flasks for a 4-day fermentation at 28°C. The resulting seed culture was, then, transferred to a fermentation tank containing 50 L millet culture medium (15 g/L millet, 5 g/L glucose, 4 g/L peptone, and 3 g/L sodium chloride, pH 7). The fermentation at 28°C was carried out for 7 days. After completion, the samples were subjected to spray drying and further treated three times with 100% methanol to obtain extracts. For the TLC dot plate experiment, a solution consisting of dichloromethane:ethyl acetate (2:1) was used. Each 250 ml bottle was washed with 3 L of eluent, and the sample was, then, centrifuged and dried. The sample components were separated and purified through chromatography using a Sephadex LH-20 column. Finally, the obtained results were compared with published Microspectral data (http://www.nmrdata.com/; Lin et al., 2016).

### Analysis of active components in the fermentation broth

The proteins in the fermentation broth were precipitated using 75% ethanol to determine their activity as ingredients. The crude extract was mixed with pre-cooled ethanol (final concentration of 75%). The resulting solution was, then, incubated at 4°C for 4 h and centrifuged at 10,000 rpm at 4°C for 20 min. The collected crude substance was resuspended in 20 mM Tris buffer (pH 6.8) before being used in subsequent experiments. To identify the active ingredients of TRM 76323^T^, freeze-dried bacteria of TRM76323^T^ were treated with dichloromethane, ethyl acetate, methanol, and water to obtain extracts (Zou et al., 2021). A 100 μl aliquot of an *A. gaisen* suspension was spread over a PDA medium. The perforated confrontation method was employed to analyze the inhibitory effects of the fermentation broth on *A. gaisen*. Holes were created in the solid medium using a sterile blue gun head, and these holes were filled with 100 μl of the fermentation broth. The medium was, then, incubated at 28°C. The inhibitory effects were assessed using the dug plate confrontation method, with the fermentation broth being tested in triplicate. *Alternaria gaisen* was activated and cultured on a PDA medium for 48 h. The PDA medium served as the control for culturing *A. gaisen*, and the compound was inoculated with a concentration of 5 μl at 1 mg/ml using the filter paper method. One plate and three treatment groups were cultivated for 7 days for SEM analysis (Zou et al., 2021).

The effects of streptochlorin (2 mg/ml) on *A. gaisen* were examined according to the filter paper method. A 5 μl aliquot of streptochlorin or chloroform (control) was added to individual pieces of filter paper. The experiment was repeated three times. To further explore how the TRM 76323^T^ fermentation broth was used to treat fragrant pears and branches that inhibited the growth and development of *A. gaisen, in vitro* experiments were conducted using fragment pears and branches that were washed three times with 75% ethanol and 5% sodium hypochlorite (Zhu et al., 2022). The lost inoculation method and sterile water treatment were used. Appropriate blank control groups were included. Samples were inoculated only with *A. gaisen* and then sprayed with the 100 μl fermentation broth after 24 h. Samples were placed in sterile bags and then incubated at 30°C for 7 days.

## Results

### Description of TRM 76323^T^

**Figure 1:**
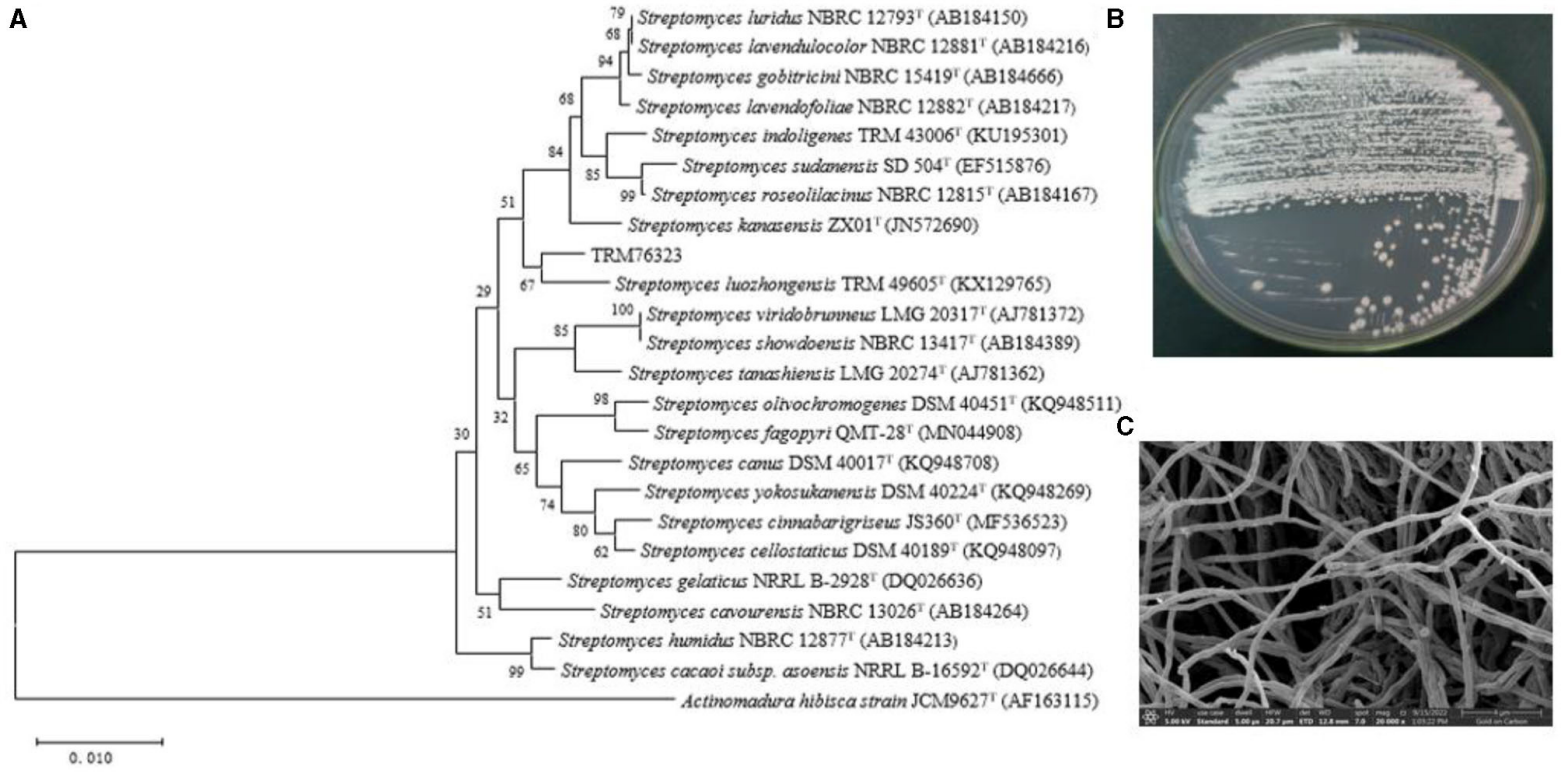
Morphological and evolutionary relationship of TRM76323. **(A)** Neighbor-joining unrooted tree based on *16S rRNA* gene sequences for strain TRM 76323^T^ and related taxa. Numbers at nodes are the bootstrap values (%) based on 1,000 resampled data sets. Bar, 0.0010 substitutions per nucleotide. **(B)** Strain morphology of TRM76323 strain cultured in ISP 4 medium. **(C)** Scanning electron micrograph of the aerial mycelium of the strain after 7 days at 28°C. Bar, 5 μm.

Strain TRM 76323^T^ exhibited good growth on ISP4, GS, ISP1, and ISP5 while showing relatively poor growth on ISP6 and NA. It was unable to grow on ISP3. The morphology of the TRM76323^T^ strain cultured in ISP 4 medium is shown in Figure 1B. Optimal growth was observed at a temperature of 28°C and a pH of 7.0, with suitable growth occurring within the temperature range of 16–37°C and pH range of 5.0–9.0. Although the cells were capable of growing in NaCl concentrations up to 5% (w/v), they exhibited the best growth in the absence of NaCl. The physiological characteristics of strain TRM 76323^T^ are presented in Table 1. Examination of a 7-day-old TRM 76323^T^ culture using an electron microscope revealed the presence of branched aerial hyphae and several long, straight, rod-shaped spores (Figure 1C). Positive results were obtained for nitrate reduction, catalase production, milk peptization, starch hydrolysis, cellulose hydrolysis, and urease production, while negative results were obtained for gelatin hydrolysis, oxidase production, melanin production, and H_2_S production.

**Table 1:** Characteristics of strain TRM 76323^T^ and the most closely related *Streptomyces* species.

**Characteristics**	**1**	**2**	**3**	**4**	**5**
**Culture characteristics:**					
Spore chain morphology	Straight	Straight	Spiral	Rectiflexibiles	Straight
Spore surface	Spiny	Smooth	Smooth	Smooth	Smooth
Substrate mycelia color	Light yellow	Light yellow	Colorless	Colorless	Light yellow
Growth on sole carbon sources (1%, w/v)	Straight	Straight	Rectiflexibiles	Straight	Straight
Production of diffusible pigments	Brown	Brown	None	None	None
D-Sorbitol	^+^	**–**	**–**	**–**	^**+**^
D-Mannitol	**–**	**–**	^+^	^+^	**–**
D-Ribose	**–**	^+^	**–**	**–**	^+^
Lactose	^+^	**–**	^+^	**–**	^+^
Maltose	**–**	**–**	^+^	**–**	^+^
L-Rhamnose	**–**	**–**	**–**	**–**	^+^
L-Arabinose	**–**	**–**	**–**	**–**	^+^
D-Xylose	**–**	**–**	**–**	**–**	^+^
Trehalose	(+)	**–**	^+^	**–**	**–**
Xanthine	ND	ND	^+^	ND	ND
Tween 20	^+^	**–**	^+^	^+^	ND
Tween 40	^+^	^+^	^+^	**–**	ND
Tween 60	^+^	^+^	^+^	**–**	ND
Tween 80	^+^	^+^	^+^	^+^	ND
**Growth at**					
45°C	**–**	**–**	^+^	^+^	^+^
Optimum pH	7-8	7-8	6-8	6- 12	6- 10
Growth with 7 % NaCl	**–**	**–**	**–**	**–**	**–**
Gelatin liquefaction	**–**	**–**	^+^	**–**	**–**
Milk coagulation peptonization	^+^	^+^	**–**	**–**	^+^
H_2_S production	**–**	^+^	**–**	**–**	**–**
Melanin production	**–**	ND	ND	**–**	ND

1, TRM76323; 2, Streptomyces luozhongensis TRM 49605^T^; 3, Streptomyces roseolilacinus NBRC 12815^T^; 4, Streptomyces kanasensis ZX01^T^; 5, Streptomyces indoligenes TRM 43006^T^. +, positive; −, negative.

### Chemotaxonomic features

The detected polar lipids were phospholipids, phosphatidylinositol, phosphatidylinositol mannoside, and phosphatidylinositol dimannoside (Supplementary Figure 1). Moreover, L,L-DAP was identified as the diagnostic cell wall diamino acid (Supplementary Figure 2A), and mannose (Man) was the main whole-cell sugar (Supplementary Figure 2B).

### Genomic and phylogenetic analyses

**Figure 2:**
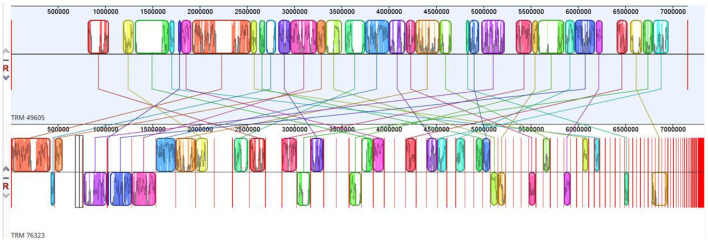
Genome-wide collinearity between TRM 76323^T^ and *Streptomyces luozhongensis* TRM 49605^T^ as determined using Geneious Prime.

The draft genome sequence for the strain TRM 76323^T^ consisted of 7,338,911 bp and comprised 127 contigs, 5 rRNAs, 103 tRNAs, and 6,330 coding sequences. The G+C content was 72.1%. Based on the comparison with strain TRM 49605, the ANI value and dDDH value for TRM 76323^T^ were 86.09 and 30.60%, respectively. A phylogenetic analysis of 16S rRNA sequences was performed using the neighbor-joining method (Figure 1A). In addition, the *atpD, gyrB, recA, rpoB*, and *trpB* gene sequences were concatenated for further analysis using the neighbor-joining (Supplementary Figure 3), maximum-likelihood (Supplementary Figure 4), and maximum-parsimony (Supplementary Figure 5) algorithms. Housekeeping genes were utilized for the multi-locus sequence analysis, which identified TRM76323^T^ as a new species. A genome-wide collinearity analysis of TRM 76323^T^ and *Streptomyces luozhongensis* TRM 49605 was conducted using Geneious Prime. Although the collinearity was relatively high, the strains were determined to be different species (Figure 2). Thirty gene clusters were detected in TRM 76323^T^, including 12 terpene, 7 NRPS, 1 T2PKS, 1 T3PKS, 1 phosphonate, and 3 T1PKS gene clusters. Among these, six gene clusters showed more than 50% similarity to known gene clusters (Supplementary Table 1). The resistance genes predicted in TRM 76323^T^ using the Comprehensive Antimicrobial Research Database (CARD) belonged to the rifampicin monooxygenase and glycopeptide resistance gene clusters and helicase-like RNA polymerase protection protein gene families, with sequence similarities of 75.52, 35.38, and 81.24%, respectively (Table 2).

**Table 2:** Strain TRM 76323^T^ resistance genes predicted using the CARD database.

**RGI criteria**	**ARO term**	**Detection criteria**	**AMR gene family**	**Drug class**	**Resistance mechanism**	**Identification of consistency %**
Strict	*Streptomyces* venezuelae rox	Protein homolog model	Rifampin monooxygenase	Rifamycin antibiotic	Antibiotic inactivation	72.52
Strict	vanH gene in vanB cluster	Protein homolog model	vanH, glycopeptide resistance gene cluster	Glycopeptide antibiotic	Antibiotic target alteration	35.38
Strict	HelR	Protein homolog model	helicase-like RNA polymerase protection protein	Rifamycin antibiotic	Antibiotic target protection	81.24

### Identification of bioactive compounds

**Figure 3:**
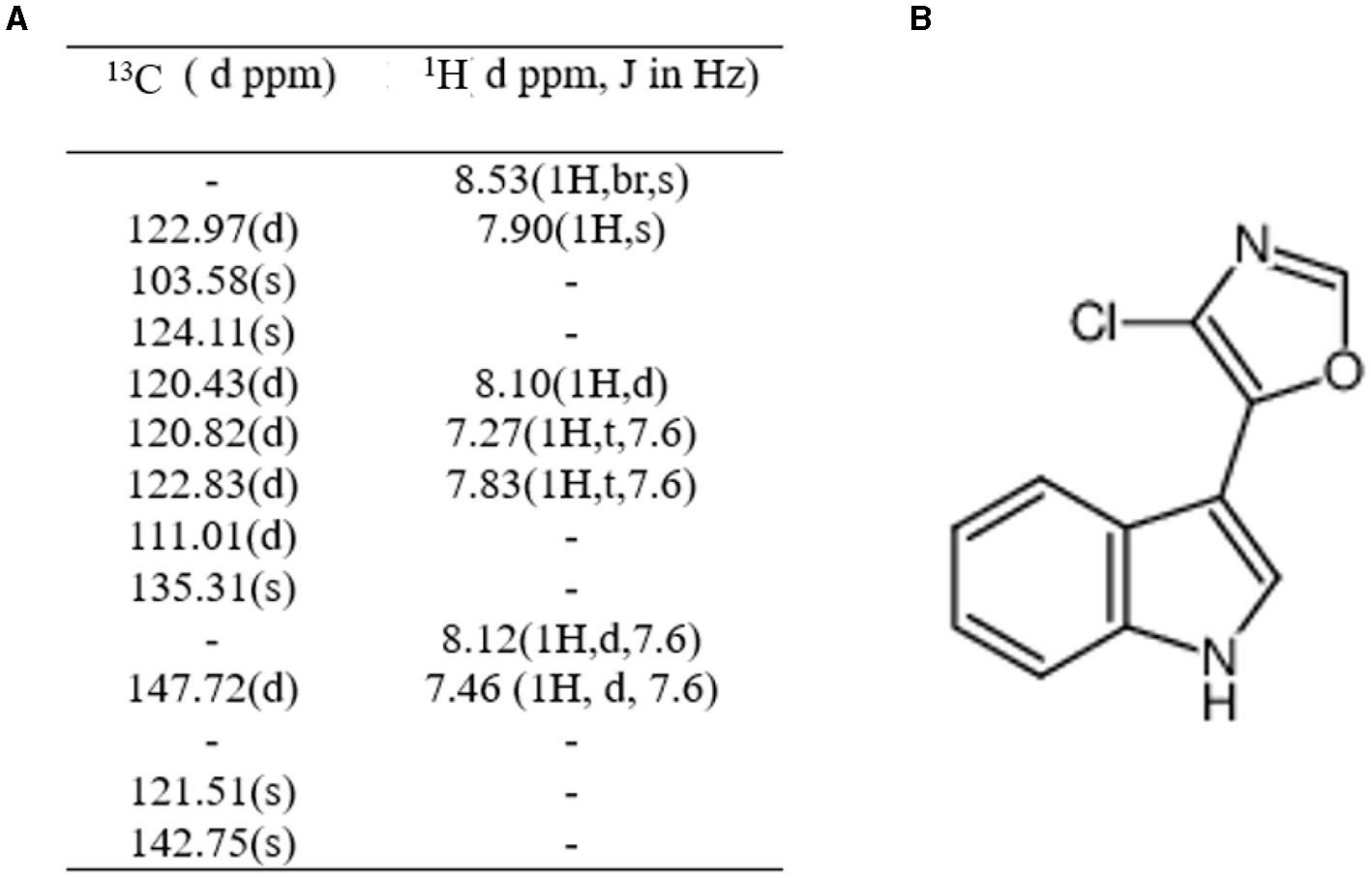
**(A)** ^1^H and ^13^C chemical shifts of the compound. **(B)** Chemical structure of streptochlorin.

One compound, streptochlorin, which exhibits tyrosinase inhibitory activity, was isolated from the methanol extract of the TRM 76323^T^ bacterial cake. Purified samples containing 12 mg of streptochlorin were obtained from the bacterial cake derived from a 50 L fermentation broth. The NMR data obtained were consistent with those obtained for the antibiotics, and their C-H data were also consistent (Supplementary Figures 6A, B). The antibiotics were identified as anti-nematode compounds based on the Combined Chemical Dictionary. Furthermore, the NMR analysis of these compounds and streptochlorin yielded very similar results (Figure 3).

### Activity screening and validation

**Figure 4:**
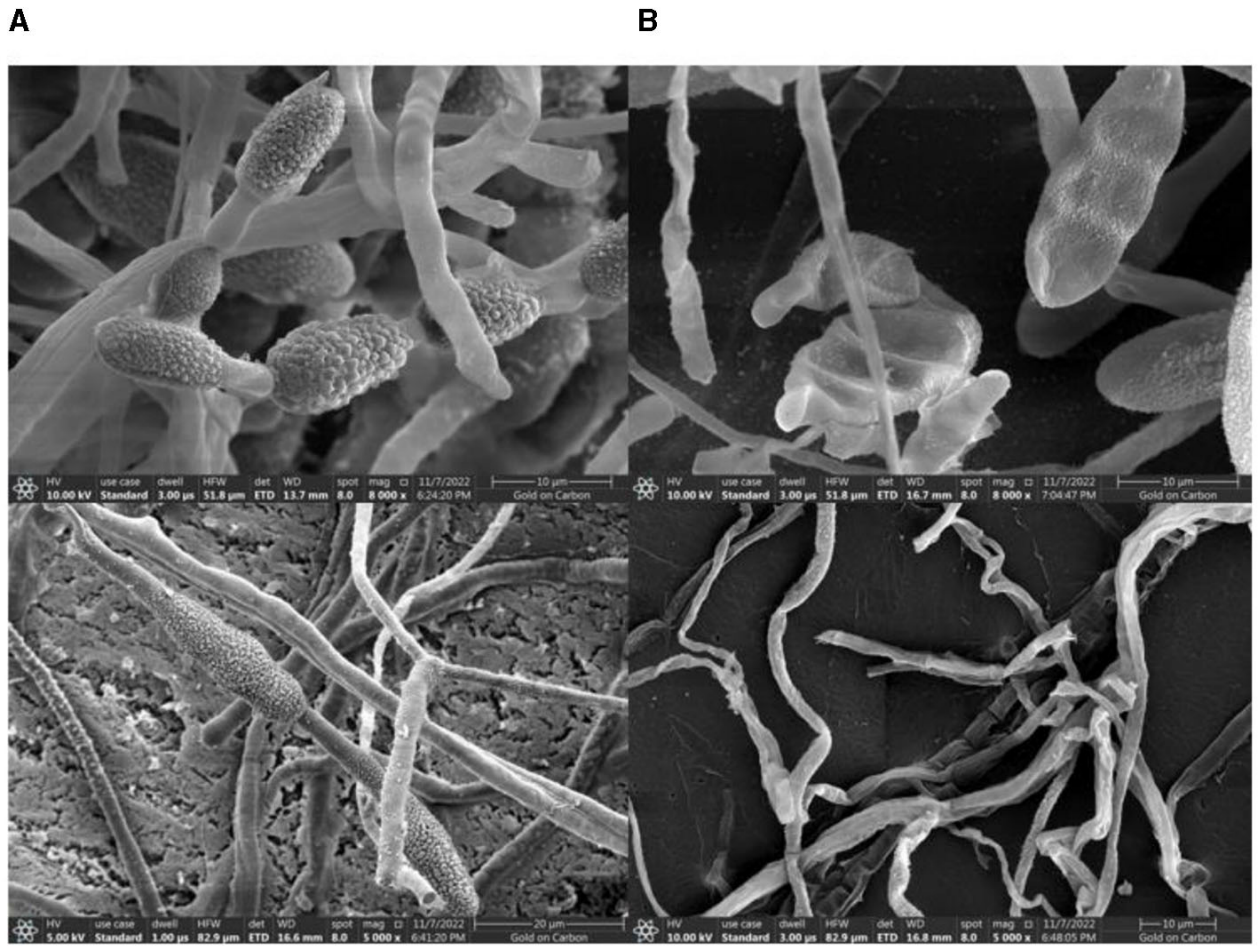
Effect of streptochlorin under SEM. **(A)** *Alternaria gaisen* under normal conditions. **(B)** *Alternaria gaisen* treated with the streptochlorin.

**Figure 5:**
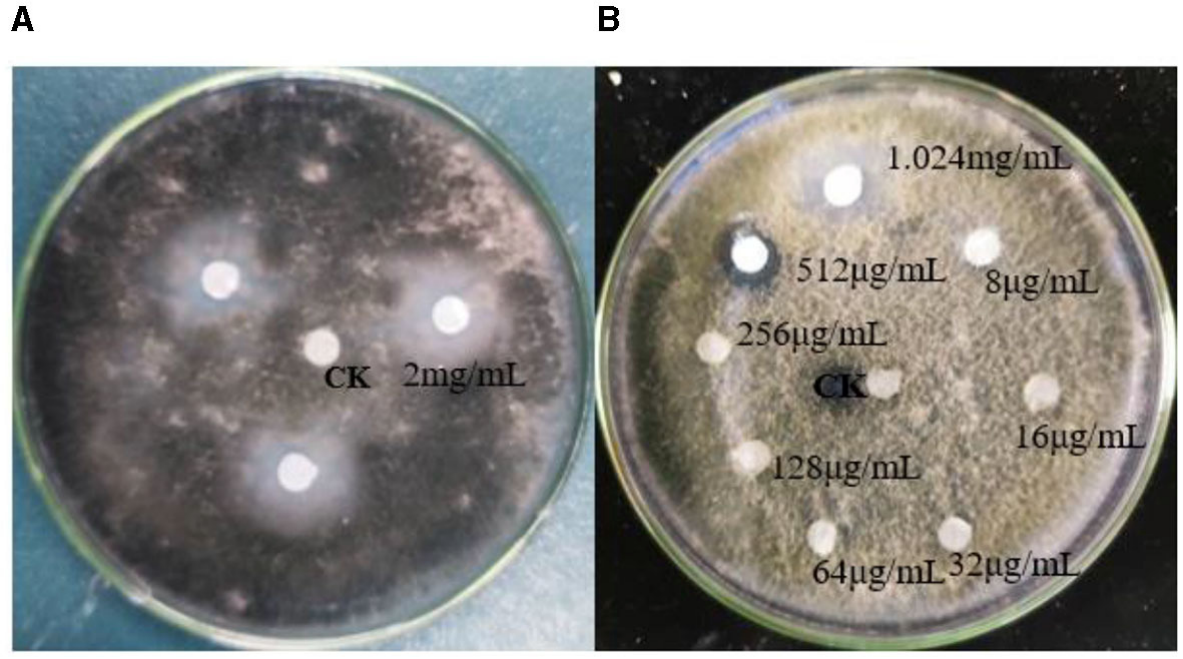
Effects of streptochlorin. **(A)** Effects of streptochlorin on *Alternaria gaisen*. **(B)** Antibacterial activity of streptochlorin at different concentrations under MIC (minimum inhibitory concentration).

**Figure 6:**
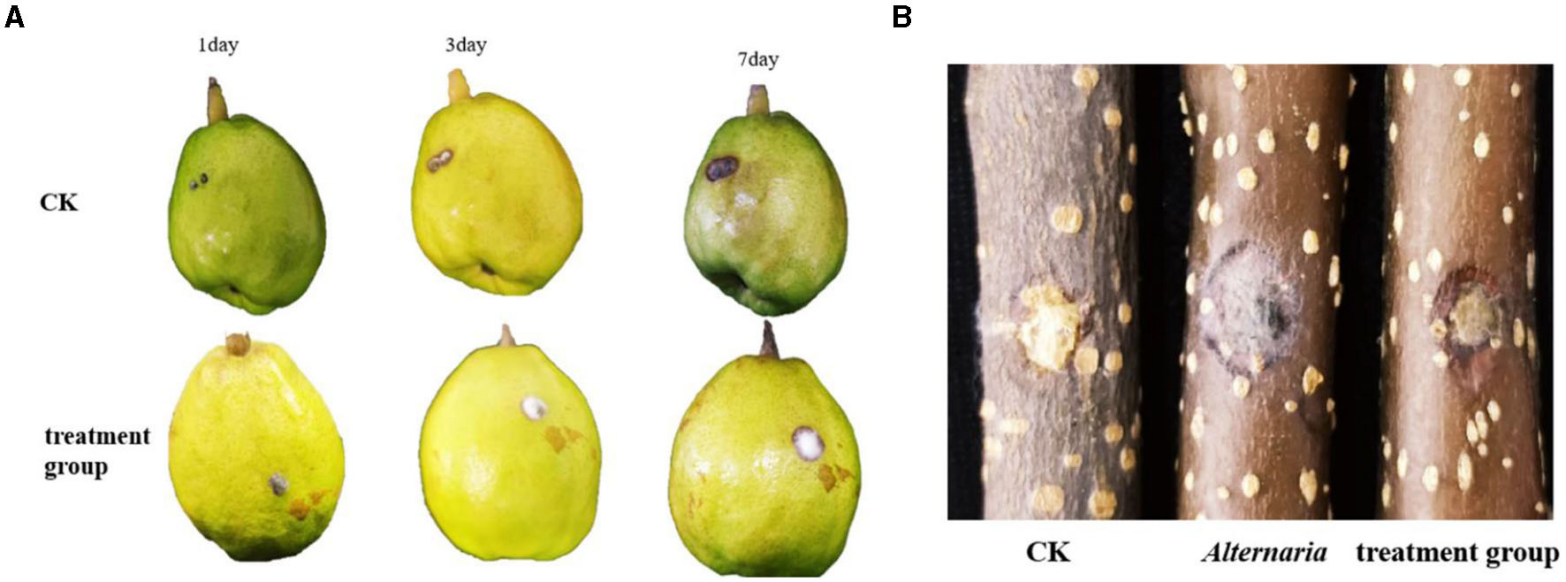
Control effect of fermentation liquid on *Alternaria gaisen*. **(A)** The inhibitory effect of streptomycin on *Streptomyces* on fruits. **(B)** Effect of streptochlorin on *Streptomyces* on tree stem. The inhibitory effect of bacteria.

The TRM 76323^T^ active components were mainly in the ethyl acetate extract (Supplementary Figure 7). The spray drying of the fermentation broth did not alter activities, implying that the components can tolerate exposure to high temperatures. The TRM 76323^T^ extracts altered the morphological characteristics of *A. gaisen* mycelia collected from the edge of the invasion zone. More specifically, the SEM indicated that the control mycelia had a smooth surface and were normal and full (Figure 4A). In contrast, the mycelia treated with TRM 76323^T^ extracts were rough, with an irregular shape and a collapsed cell wall (Figure 4B). The activity of streptochlorin was tested at a concentration of 2 mg/ml; the bacteriostatic effect resulted in an inhibition of ~1.5 ± 0.22 cm (Figure 5A). The minimum bacteriostatic concentration determined using the MIC method was 512 μg/ml (i.e., good bacteriostatic activity; Figure 5B). The effects of the TRM 76323^T^ fermentation broth on fragrant pear fruits infected with *A. gaisen* were investigated. The fragrant pear fruit inoculated with *A. gaisen* had black spot disease symptoms and the incubation was for 7 days. Branches infected with *A. gaisen* also showed symptoms of black spot disease over an incubation period of 7 days. However, treating the affected fragrant pear fruit and branches with the TRM 76323^T^ fertilization broth inhibited the severity of the black spot disease symptoms over the incubation period of 7 days (Figures 6A, B).

## Discussion

Pear black spot, caused by *A. gaisen*, is a destructive disease that negatively impacts pear tree yield. The use of *Streptomyces*-based biological control strategy presents a viable alternative to chemical fungicides as previous research has shown promising results (Song et al., 2020; Yuan et al., 2021). However, there is a scarcity of biological control strains with antagonistic activity against pear black spot disease. Hence, this study focuses on isolating strain TRM76323^T^ from *Tamarix* and evaluating its ability to inhibit the growth of pear black spot through activity tracking. Strain TRM76323^T^ produces streptochlorin, which effectively inhibits the hyphal growth of *A. gaisen* and induces hyphal malformation, aligning with previous studies. Overall, these findings highlight the potential of strain TRM76323^T^ as a promising biological control resource for combating pear black spot disease. The survival of biocontrol strains in plants is crucial for their agricultural application. Microorganism-produced compounds can negatively impact fungal cells' growth and morphology, potentially leading to cell death (Bowman and Free, 2006). Notably, the culture filtrate of strain TRM76323^T^ exhibits antifungal activity against *A. gaisen*, indicating the presence of antibacterial compounds. Numerous studies have demonstrated the contribution of microorganism secondary metabolites to plant resistance (Piasecka et al., 2015). In conclusion, these findings suggest that TRM76323^T^ can induce antifungal defense in pears primarily by regulating the biosynthesis pathway of secondary metabolites.

## Conclusion

In this study, on the basis of polyphasic classification and identification, physiological and biochemical indicators, chemical indicators, and genome sequence comparisons, TRM 76323^T^ was identified as a new species. Its inhibitory activities against *A. gaisen* were verified, and its active components were identified. Microscopic examinations and *in vivo* experiments confirmed the inhibitory effects of TRM 76323^T^ on *A. gaisen* at the micro- and macro-levels. Furthermore, streptochlorin was purified, with the subsequent analysis, revealing that its minimum inhibitory concentration was 512 μg/ml. In summary, our results showed not only the biosynthetic potential of strain TRM76323^T^ for the biopharmaceutical field but also its distinguishable phenotypic and genotypic properties, confirming that it represents a novel species of the genus *Streptomyces* for which the name *Streptomyces sp*. nov. is proposed. The findings of this study form the basis of future research conducted to increase the utility of strain TRM 76323^T^ for controlling agriculturally important plant diseases.

### Description of *Streptomyces* sp.nov.

Strain TRM76323 can grow well on ISP1, ISP4, ISP5, and Gause's medium and can produce abundant spores after 7 days of cultivation. However, the growth on ISP6 and NA media is relatively poor, and it cannot grow on ISP3 medium. The optimal pH is 7.0, the optimal growth temperature is 28°C, and the strain can grow at a concentration of 5% (w/v) NaCl, with the best growth at a salt concentration of 0%. Strain TRM76323 can use D-sorbitol, lactose, and trehalose as the only carbon sources. Nitrate reduction, catalase, milk coagulation and peptonization, starch hydrolysis, cellulose hydrolysis, and urease production all obtained positive results, but gelatin hydrolysis, oxidase production, and melanin production all obtained negative results. TRM76323 was able to grow on media supplemented with Tween 20, Tween 40, Tween 60, and Tween 80, producing hydrolysis loops, indicating that the strain has lipase activity. The polar lipids of strain TRM76323 are PI (phosphatidylinositol), PIM (phosphatidylinositol mannoside), PIDM (phosphatidylinositol diamandoside), and PLS (phospholipid). The amino acid component of the cell wall is Diaminopimelic acid; TRM76323 whole-cell hydrolytic sugar component contains mannose, TRM76323, and S. The DNA–DNA hybridization (DDH) result of *Streptomyces luozhongensis* TRM49605^T^ is 30.60%, which is far below from the threshold defined species by 70%, and the ANI value is 86.09%, which is lower than the threshold describing prokaryote species by 95%, indicating that TRM76323 and *S. luozhongensi*s TRM49605^T^ are different species. Strain TRM76323 was identified as a new species of *Streptomyces* through polyphasic classification. This study is activity-oriented, with the goal of preventing and controlling pear black spot disease. Using modern separation and purification techniques, the active component of strain TRM76323 was determined to be located in the ethyl acetate phase using the plate confrontation method. According to the HPLC method, the active product was separated, and the active product was identified as streptochlorin, so the minimum inhibitory concentration was 512 μg/ml. This study analyzes the antagonistic effect of the new species of *Streptomyces* sp. TRM76323 on pear black spot disease, which can more effectively carry out the prevention and control of pear black spot disease, reduce economic losses, and promote sustainable development of agriculture to lay the foundation for the healthy development of the pear industry and ensure the economic stability of the pear region. As a new means of disease control, microbial lead compound is characterized by safety, economy, and no resistance to pests and diseases. It provides a new vision and technical basis for sustainable agricultural development strategy and provides a theory for the formulation of pathogen control measures.

## Data availability statement

The datasets presented in this study can be found in online repositories. The names of the repository/repositories and accession number(s) can be found at: 菌株保藏于 CCTCC M2023092.

## Author contributions

Y-hC: Writing – original draft, Validation. YW: Validation, Investigation, Data curation, Writing – original draft. QL: Conceptualization, Supervision, Writing – review & editing. ZX: Supervision, Writing – review & editing. JW: Conceptualization, Writing – review & editing. X-XL: Funding acquisition, Methodology, Supervision, Writing – review & editing.
