# Supplementary material for: Streptomyces tamarix sp. nov.: antagonism against Alternaria gaisen producing streptochlorin, isolated from Tamarix root soil
Source: Front Microbiol. 2023 Nov 21;14:1273842. doi: 10.3389/fmicb.2023.1273842 (PMC10702757; doi:10.3389/fmicb.2023.1273842)
Supplement: Supplementary file 1 [file Data_Sheet_1.docx]

Supplementary Material


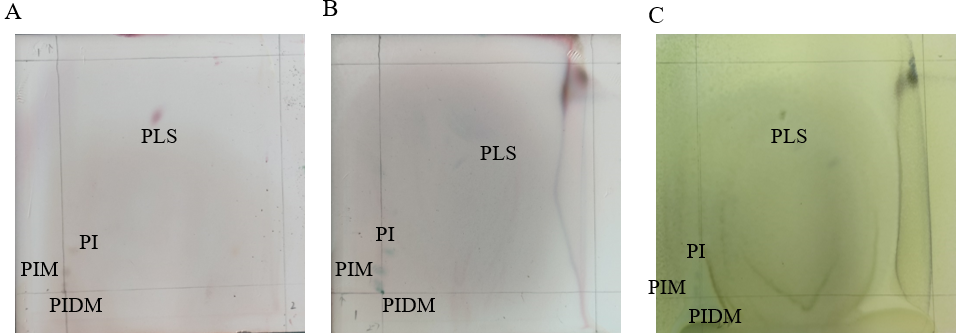


**Supporting Figure 1. (A)** Ninhydrin. **(B)** Anisaldehyde. **(C)** Molybdophosphoric acid. Strain TRM 76323^T^polar lipid composition separated by two-dimensional TLC. PI, phosphatidylinositol; PIM, phosphatidylinositol mannoside; PIDM, phosphatidylinositol dimannoside; PLS, phospholipid.


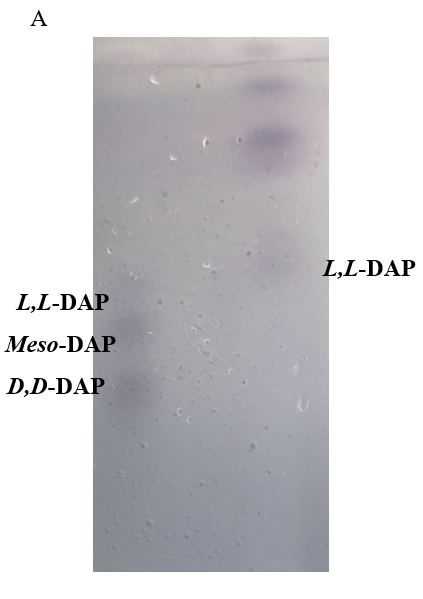

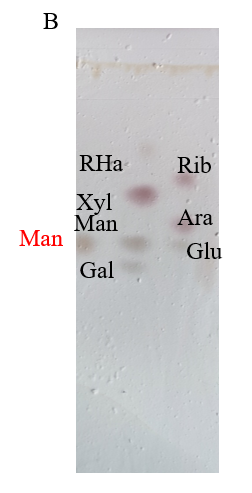


**Supporting Figure 2 (A**) The main amino acid type in TRM 76323^T^ is L,L-DAP. **(B)** The main whole-cell sugar in TRM 76323^T^ is Man.

##
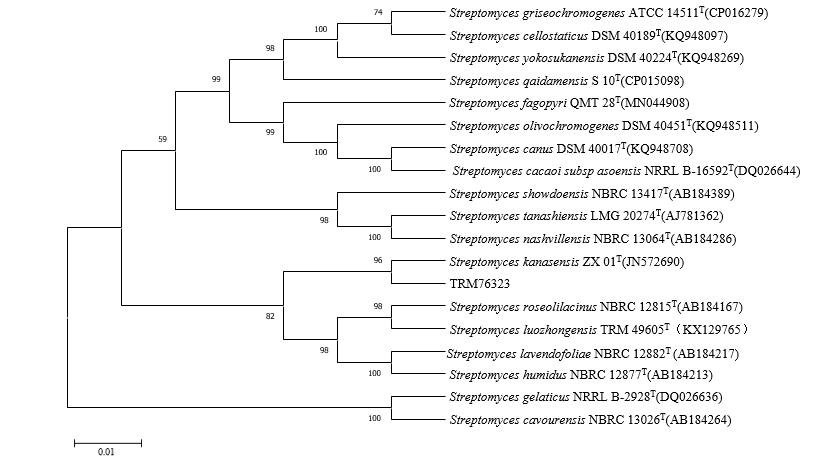


## Supporting Figure 3 Neighbor-joining phylogenetic tree based on concatenated partial sequences of the housekeeping genes *atpD*, *gyrB*, *recA*, *rpoB*, and *trpB*. The relationships between strain TRM 76323^T^ and the type strains of phylogenetically closely related species from the genus *Streptomyces* were analyzed. Numbers at nodes are bootstrap values (%) calculated using 1,000 resampled data sets. Bar, 0.01 substitutions per nucleotide.


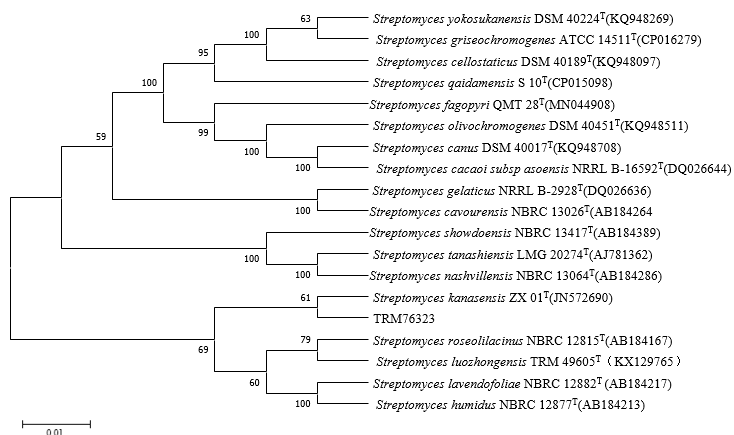


**Supporting Figure 4** Maximum-likelihood phylogenetic tree based on concatenated partial sequences of the housekeeping genes *atpD*, *gyrB*, *recA*, *rpoB*, and *trpB*. The relationships between strain TRM 76323^T^ and the type strains of phylogenetically closely related species from the genus *Streptomyces* were analyzed. Numbers at nodes are bootstrap values (%) calculated using 1,000 resampled data sets. Bar, 0.01 substitutions per nucleotide.


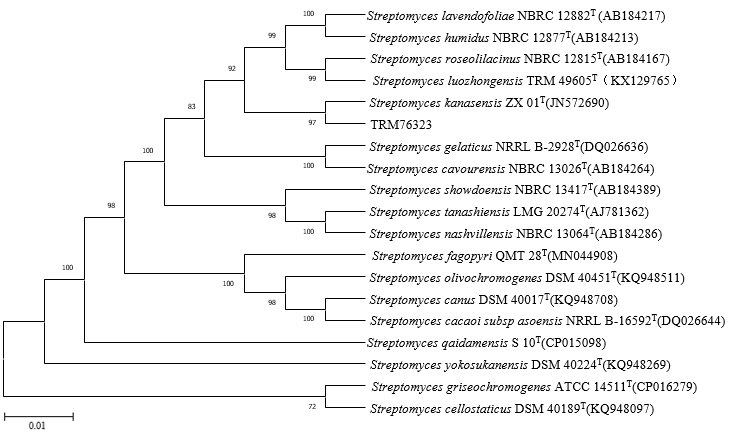


**Supporting Figure 5** Maximum-parsimony phylogenetic tree based on the concatenated partial sequences of the housekeeping genes *atpD*, *gyrB*, *recA*, *rpoB*, and *trpB*. The relationships between strain TRM 76323^T^ and the type strains of phylogenetically closely related species from the genus *Streptomyces* were analyzed. Numbers at nodes are bootstrap values (%) calculated using 1,000 resampled data sets. Bar, 0.01 substitutions per nucleotide.


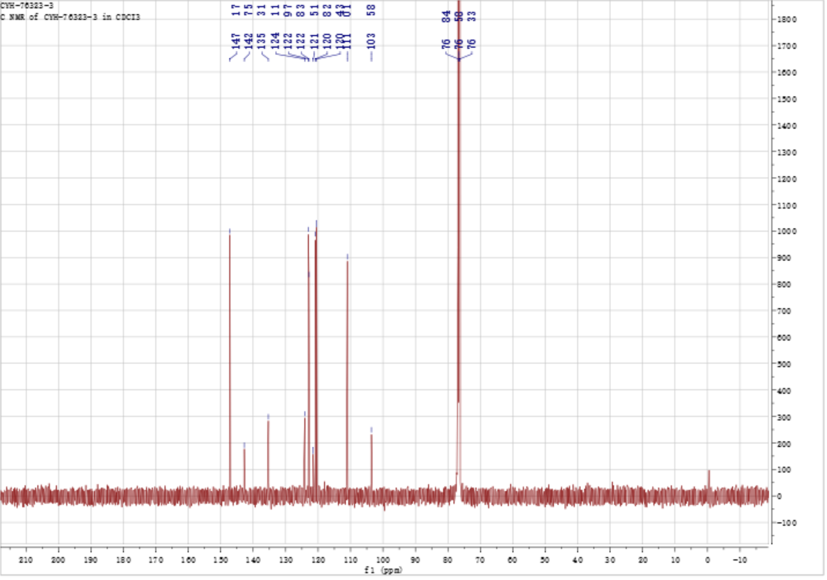


**Supporting Figure 6 (A)** Nuclear magnetic C spectrum of TRM 76323^T^. Chloroform was used as the solvent.


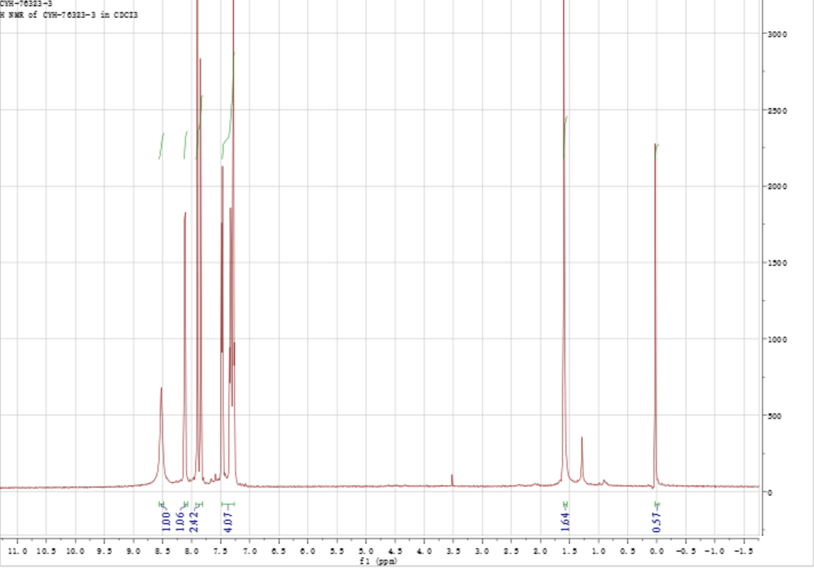


**Supporting Figure 6 (B)** Nuclear magnetic H spectrum of TRM 76323^T^. Chloroform was used as the solvent.


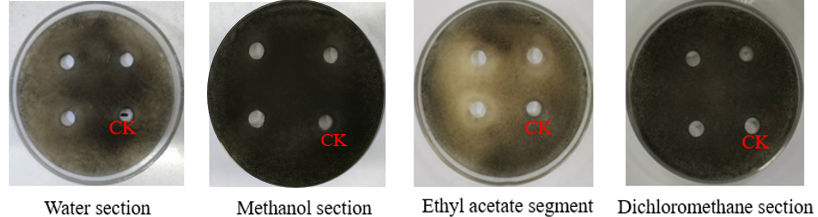


**Supporting Figure 7** Antibacterial effects of TRM 76323^T^ extracts prepared using different solvents.

**Supplementary Table 1 TRM76323 biosynthetic gene cluster**

| Region | Type | Most similar known cluster | Similarity | % |
| --- | --- | --- | --- | --- |
| 1 | T2PKS | hiroshidine | Polyketide | 89 |
| 2 | PKS-like,lanthipeptide-class-i | rustmicin | Polyketide:Iterative type I | 6 |
| 3 | siderophore | desferrioxamin B | Other | 100 |
| 4 | RiPP-like |  |  |  |
| 5 | butyrolactone,betalactone | A-201A | Other | 6 |
| 6 | NRPS | neocarzinostatin | Polyketide:Iterative type I + Polyketide:Enediyne type I | 6 |
| 7 | T1PKS |  |  |  |
| 8 | hglE-KS,phosphonate,  lanthipeptide-class-iv | dehydrofosmidomycin | Other | 23 |
| 9 | terpene | BE-43547A1 BE-43547A2 BE-43547B1 BE-43547B2 BE-43547B3 BE-43547C1 BE-43547C2 | NRP:Cyclic depsipeptide + Polyketide:  Modular type I | 20 |
| 10 | terpene | hopene | Terpene | 15 |
| 11 | lanthipeptide-class-iii | SapB | RiPP:  Lanthipeptide | 100 |
| 12 | NRPS-like | kirromycin | NRP + Polyketide:  Modular type I + Polyketide:  Trans-AT type I | 3 |
| 13 | terpene | geosmin | Terpene | 100 |
| 14 | lanthipeptide-class-i | akaeolide | Polyketide | 12 |
| 15 | NRPS,T1PKS,other | ecumicin | NRP | 15 |
| 16 | betalactone |  |  |  |
| 17 | ladderane,NRPS | RP-1776 | Polyketide + NRP:Cyclic depsipeptide | 20 |
| 18 | NRPS,NRPS-like | soraphen A | Polyketide:  Modular type I | 15 |
| 19 | ectoine | ectoine | Other | 100 |
| 20 | terpene |  |  |  |
| 21 | terpene | isorenieratene | Terpene | 100 |
| 22 | siderophore | ficellomycin | NRP | 12 |
| 23 | terpene,other,phenazine,  butyrolactone | marinophenazine A / phenaziterpene A | Other:Phenazine | 30 |
| 24 | T1PKS | zorbamycin | NRP:Glycopeptide + Polyketide:  Modular type I + Saccharide:  Hybrid/tailoring | 6 |
| 25 | thioamitides |  |  |  |
| 26 | butyrolactone | prejadomycin / rabelomycin / gaudimycin C / gaudimycin D / UWM6 / gaudimycin A | Polyketide:  Type II + Saccharide:  Hybrid/tailoring | 6 |
| 27 | NRPS,lanthipeptide-class-ii |  |  |  |
| 28 | thiopeptide,LAP | lactazole | RiPP:Thiopeptide | 44 |
| 29 | terpene | hopene | Terpene | 69 |
| 30 | T3PKS | napyradiomycin A80915C | Terpene Polyketide:  Type III | 6 |
